# Supplementary material for: Stress-testing the resilience of the Austrian healthcare system using agent-based simulation
Source: Nat Commun. 2022 Jul 23;13:4259. doi: 10.1038/s41467-022-31766-7 (PMC9308034; doi:10.1038/s41467-022-31766-7)
Supplement: Supplementary file 1 — Supplementary Information [file 41467_2022_31766_MOESM1_ESM.pdf]

Supplemental Information

A Matching of patient contact data to opening hours

To assess the patient contact capacity of physicians, we obtained the permission to scrape data about opening hours from the platform `www.herold.at` for Austrian physicians in March 2020. The scraped data includes a physician's unique ID, speciality, municipality, address, information about whether s/he is a panel physician and opening times for every day of the week. The opening hour data includes a total of 49,562 unique (physician, specialisation, municipality) combinations,

Before probabilistically matching the opening hour data to the patient contact data, we perform a number of cleaning and data enrichment steps: We only include panel physicians in our analysis, reducing the number of physicians to 7,831. We further exclude all physicians that do not indicate any opening hours (1,137 physicians). Physicians that indicate opening hours only "by appointment" on a given day are assumed to be open for 2 hours on that day. If there is more than one entry of the same physician but with different opening hours (29 entries in total), we keep the entry with the longer opening hours. We then match the 29 specialities given in the opening hours data set (see table S1) to the 13 specialities given in the patient contact data (see Table 1). We drop 9 specialities – such as medical and chemical laboratory diagnostics – that are not corresponding to any of the specialities in the patient contact data (90 physicians). This leaves us with a total of 6,604 unique physicians.

We then probabilistically assign the opening hour data to the patient contact data following a two-step process: We first look for all instances where there is only a single physician with a given speciality in a given district in both data sets and match these physicians. This results in 1,233 direct matches. For the remaining physicians, we calculate the vector of proportional opening times $\mathbf{v}$ for every day of the week: if a physician has a total of $h = 32$ opening hours of which 8 fall on Mondays, 8 on Tuesdays, 8 on Wednesdays, 4 on Thursdays and 4 on Fridays, this results in an opening hour vector of $\mathbf{v} = (8/h, 8/h, 8/h, 4/h, 4/h, 0/h, 0/h) = (0.25, 0.25, 0.25, 0.125, 0.125, 0, 0)$. We also calculate the vector of proportional patient contacts $\mathbf{w}$ for the physicians in the patient contact data set: if a physician has a total of $n = 10,000$ patient contacts throughout the year, of which 1,500 occurred on Mondays, 1,500 on Tuesdays, 3,000 on Wednesdays, 2,000 on Thursdays and 2,000 on Fridays, this results in a patient contact vector of $\mathbf{w} = (1500/n, 1500/n, 3000/n, 2000/n, 2000/n, 0/n, 0/n) = (0.15, 0.15, 0.3, 0.2, 0.2, 0, 0)$.

We then assign physicians with a given speciality and within a given municipality based on the difference between their opening hours and patient contacts by minimizing $E = \sum_{i=1}^{7} |v_i - w_i|$. We also introduce a threshold $\epsilon = 0.5$ and only match two physicians if $E \leq \epsilon$. This results in 2,891 additional matches (2,537 matches if $\epsilon = 0.3$ and 3,055 matches if $\epsilon = 0.7$). At the end of the procedure, we successfully assigned capacities to 4,288 physicians from the opening hour data set to the patient contact data set, leaving 2,406 physicians (35.9%) in the opening hour data set and 5,292 physicians (55.2%) in the patient contact data set unmatched. We perform a left join of the physicians from the patient contact data and the matched opening hour information, resulting in a total of 9,580 physicians of which 44.8% have opening hour information.

Inspection of the distribution of opening hours of matched and unmatched physicians (see SI) shows that there is a slight bias towards unmatched physicians having fewer opening hours. This difference is significant ($p < 0.05$, two-sided t-test of the opening hour distributions) for general practitioners ($\bar{h}_{\text{matched}} = 20.3 \pm 4.7$ h, $\bar{h}_{\text{unmatched}} = 19.7 \pm 6.8$ h, mean $\pm$ standard deviation), urologists ($\bar{h}_{\text{matched}} = 20.5 \pm 5.0$ h, $\bar{h}_{\text{unmatched}} = 18.6 \pm 7.4$ h) and dermatologists ($\bar{h}_{\text{matched}} = 21.4 \pm 5.1$ h, $\bar{h}_{\text{unmatched}} = 19.6 \pm 5.0$ h). We see a similar trend in the distributions of patient contacts (see SI), as unmatched physicians tend to have fewer yearly patient contacts. This difference is significant ($p < 0.05$) for general practitioners ($\bar{n}_{\text{matched}} = 16054 \pm 7881$ h, $\bar{n}_{\text{unmatched}} = 10770 \pm 6688$ h), internists ($\bar{n}_{\text{matched}} = 15032 \pm 26184$ h, $\bar{n}_{\text{unmatched}} = 7165 \pm 6201$ h), gynaecologists ($\bar{n}_{\text{matched}} = 7125 \pm 6579$ h, $\bar{n}_{\text{unmatched}} = 5608 \pm 3723$ h), ophtalmologists ($\bar{n}_{\text{matched}} = 11330 \pm 10254$ h, $\bar{n}_{\text{unmatched}} = 8174 \pm 5767$ h), otolaryngologists ($\bar{n}_{\text{matched}} = 8925 \pm 4903$ h, $\bar{n}_{\text{unmatched}} = 7667 \pm 4463$ h), dermatologists ($\bar{n}_{\text{matched}} = 13582 \pm 11496$ h, $\bar{n}_{\text{unmatched}} = 9891 \pm 5996$ h), orthopaedists ($\bar{n}_{\text{matched}} = 24376 \pm 33647$ h, $\bar{n}_{\text{unmatched}} = 13062 \pm 14792$ h) and radiologists ($\bar{n}_{\text{matched}} = 25616 \pm 27148$ h, $\bar{n}_{\text{unmatched}} = 13799 \pm 10436$ h). The matching rate (number of matched physicians / total number of physicians) is best for general practitioners (55.1%) and worst for internists (20.7%) (see SI).

For the unmatched physicians, we impute the opening hour information in the following way: for every unmatched physician, we look for all successfully matched physicians with the same specialisation and similar number of patients. We first look for all physicians that have the same number of annual patients ± 10. If no physicians are found, we increase the search interval to ± 100 or ± 1000 annual patients. We then calculate the average opening hours for each day of the week

for these successfully matched physicians and use this information to impute the missing opening hour information for the unmatched physicians.

opening hours	patient contacts	n
general medicine	general practitioner	3552
internal medicine	internist	423
obstetrics and gynaecology	gynaecologist	395
ophtalmology and optometry	ophtalmology	363
paediatric medicine	paediatrician	258
otorhinolaryngology, ophthalmology and angiology	otorhinolaryngologist, ophthalmologist and angiologist	238
orthopaedics and orthopaedic surgery	orthopaedist	232
dermatology and sexually transmitted diseases	dermatologist	226
radiology	radiologist	213
urology	urologist	178
psychiatry	psychiatrist	174
pulmonology and pneumology	internist	148
surgery	surgeon	118
neurology	neurologist	67
medical and chemical laboratory diagnostics	–	32
childhood and adolescent psychiatry	–	19
physical medicine and general rehabilitation	–	17
trauma surgery	surgeon	17
clinical pathology and molecular pathology	–	8
clinical microbiology and hygienics	–	5
dental, oral and maxillo-facial surgery	–	3
plastic, reconstructive, and aesthetic surgery	–	3
childhood and adolescent psychiatry and psychotherapy	–	2
neurosurgery	neurosurgeon	2
anesthesiology and intensive care medicine	–	1

Supplementary Table 1: Matching of data sets. Mapping of physician's specialities between the opening hour data set and the patient contact data set.

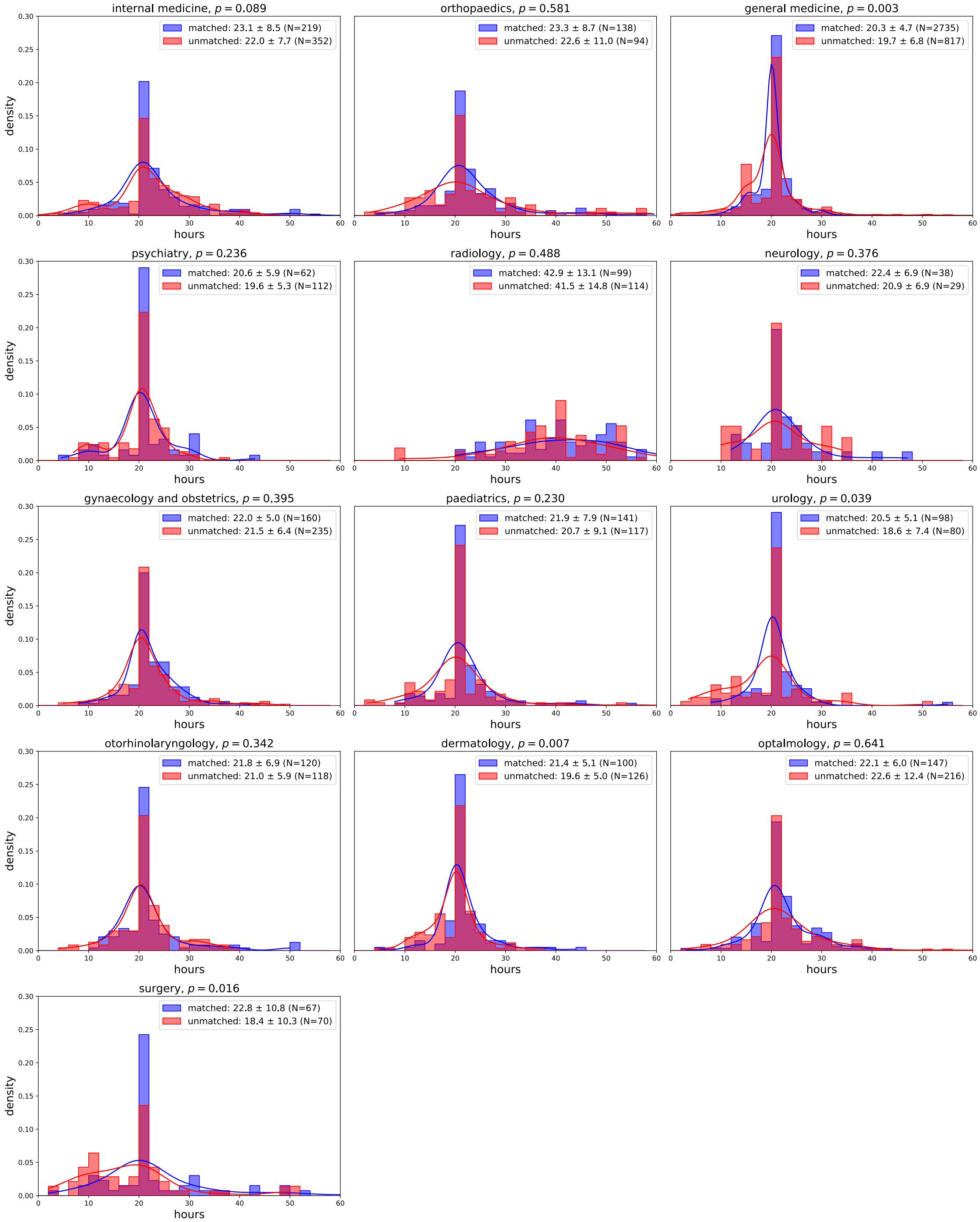

Supplementary Figure 1: Opening hours distribution. Distribution of opening hours of matched (blue) and unmatched (red) physicians from the opening hour data set. P-values from two-sided independent t-tests between the distributions are indicated for each speciality.

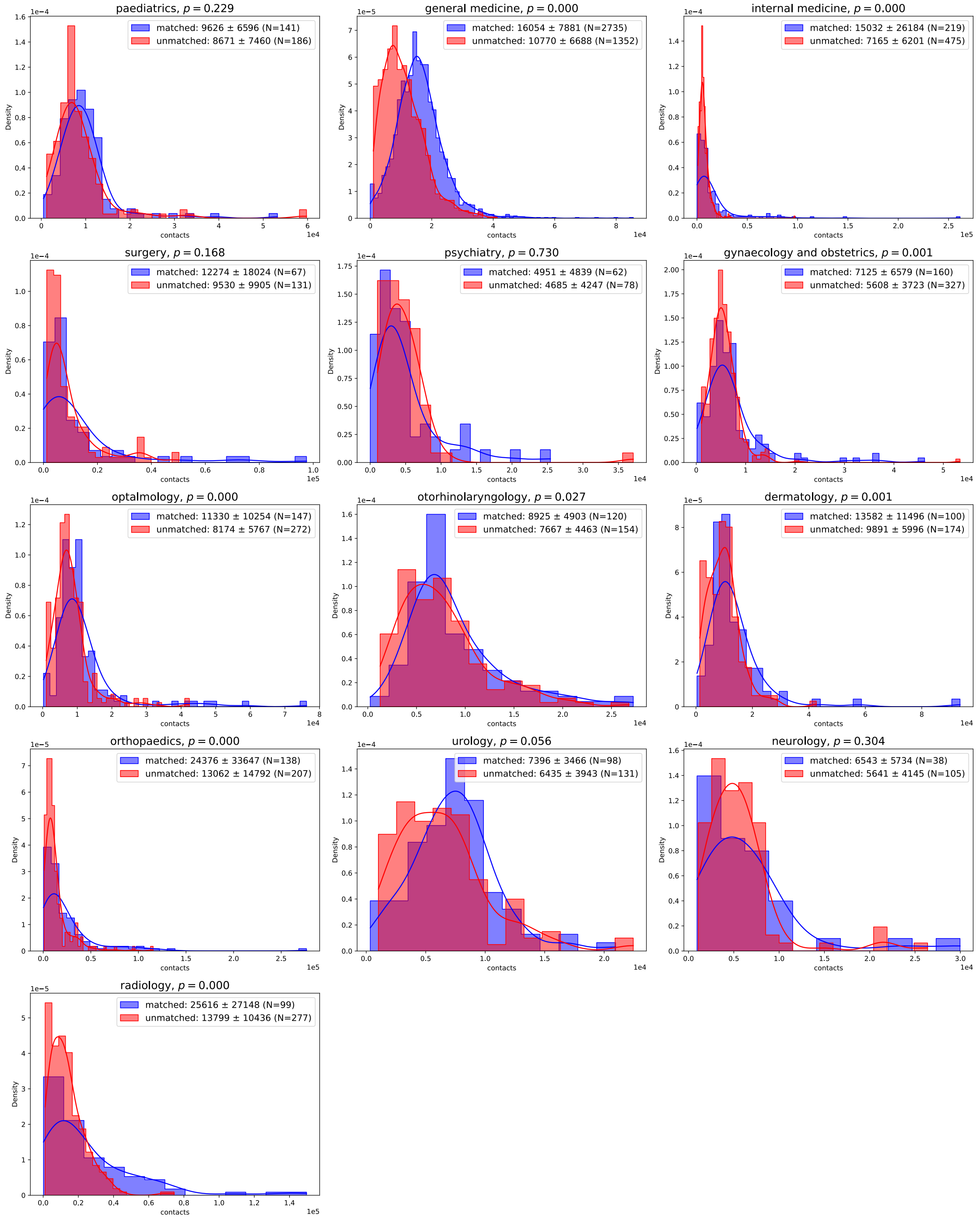

Supplementary Figure 2: Patient contacts distribution. Distribution of patient contacts of matched (blue) and unmatched (red) physicians from the patient contact data set. P-values from two-sided independent t-tests between the distributions are indicated for each speciality.

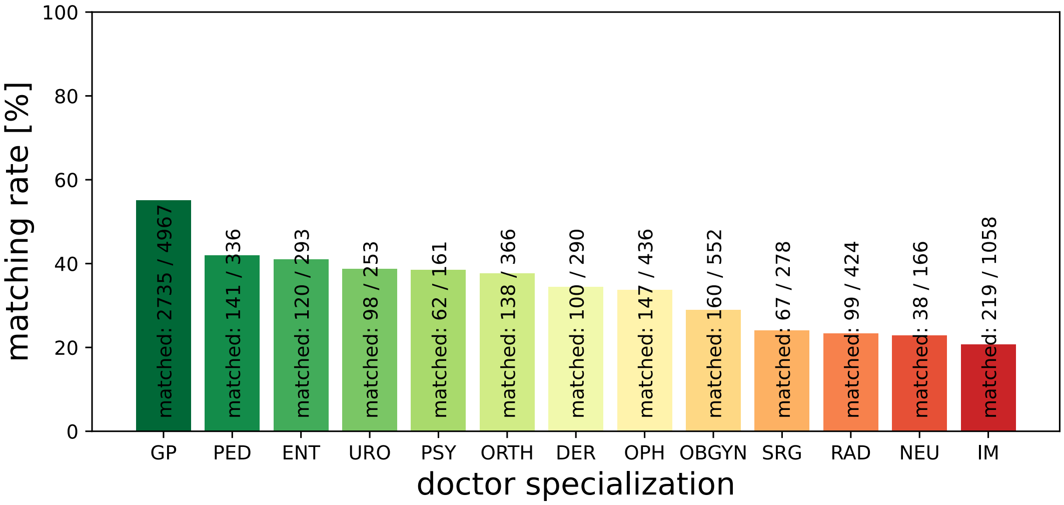

Supplementary Figure 3: Matching rates. % rates of matched physicians from the patient contact data set for each of the 13 specialities.

B Progress of resilience indicators in states

In Supplementary Figure 4 we show nationwide averages of resilience indicators. In addition to regional resilience of the healthcare system, we also investigate the individual risk and benefit that a physician contributes to the system. To this end, for each specialty j we average the individual risk (risk_j) and benefit ($\mathrm{benefit}_j$) scores over all federal states to obtain nationwide results. Supplementary Figure 4 (b) shows the mean and standard deviation for each specialty averaged over all federal states.

Supplementary Figures 5 to 16 show state-level simulation results for the continuous physician removal process for different specialties (except GP). Parts a) show the cumulative

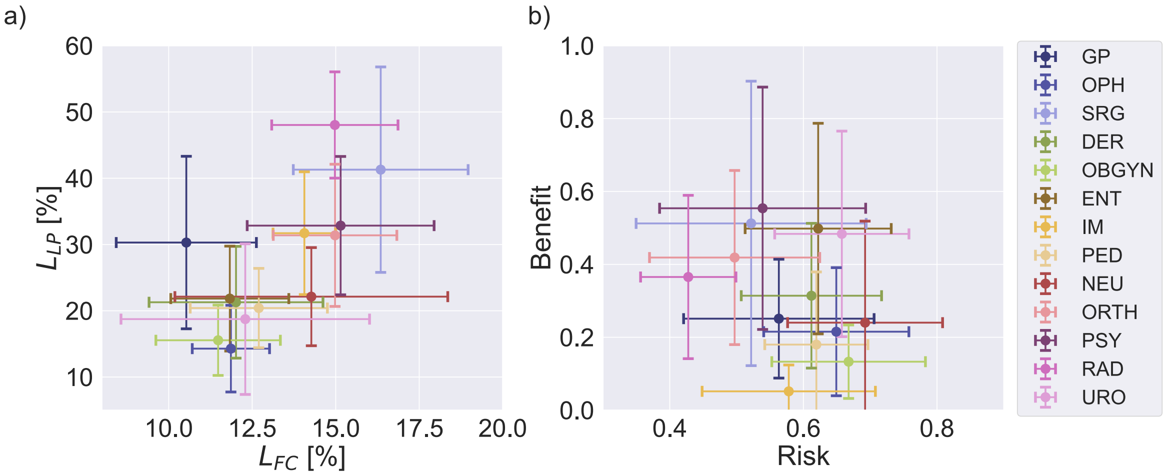

Supplementary Figure 4: Country-level specialty specific simulation results. a) shows specialty specific risk and benefit scores averaged over all states, b) shows state averaged percentages of physicians that can be removed before critical limits for lost patients and free capacity are reached. For a list of abbreviations of specialists see Table 1 in the main text.

relative lost patients in states, parts b) show gradually filled up free capacity in states.

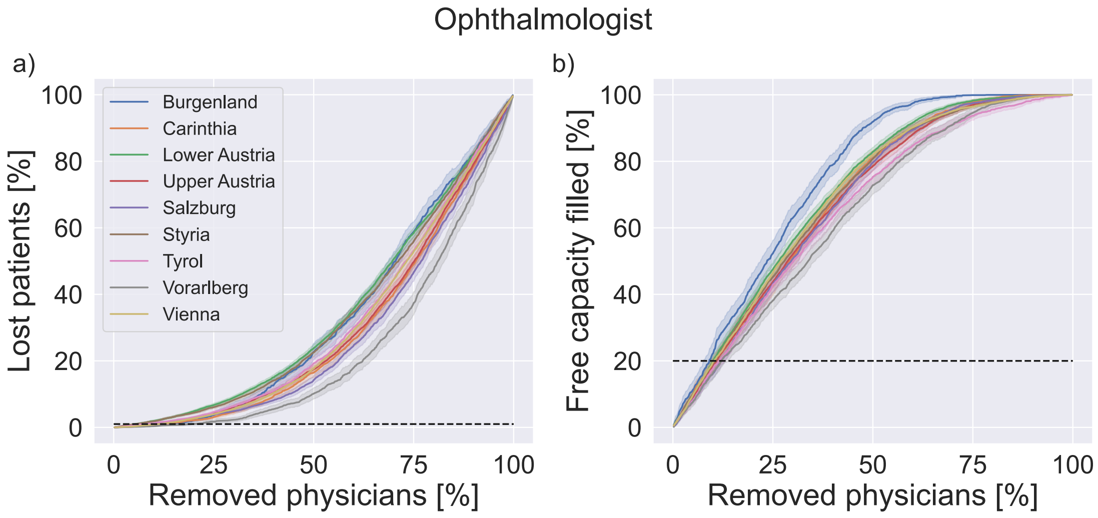

Supplementary Figure 5: Resilience indicators for ophthalmology. Lost patients and free capacity as a function of removed physicians for ophthalmologists. Lines and error bands show mean and standard deviation, respectively.

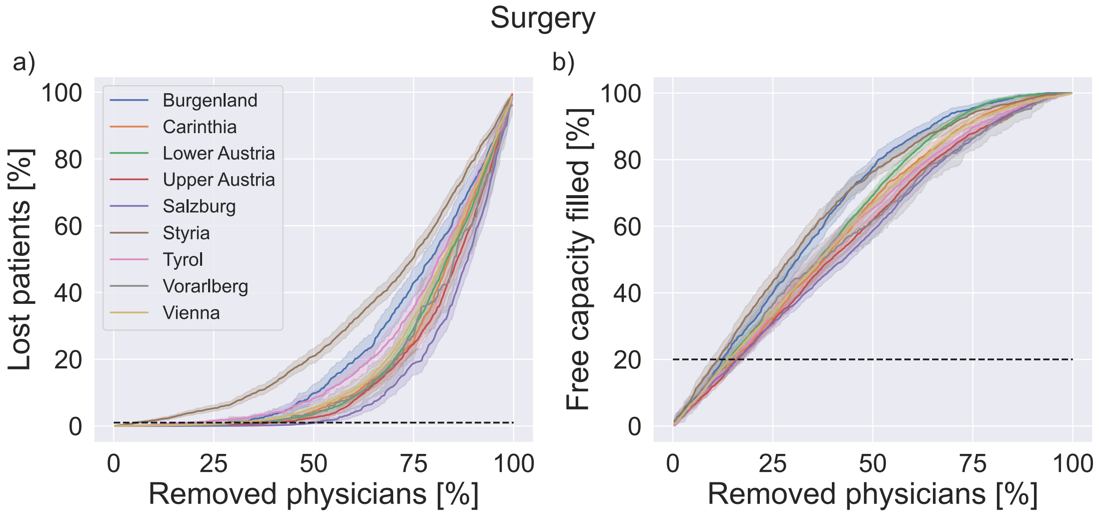

Supplementary Figure 6: Resilience indicators for surgery. Lost patients and free capacity as a function of removed physicians for surgery. Lines and error bands show mean and standard deviation, respectively.

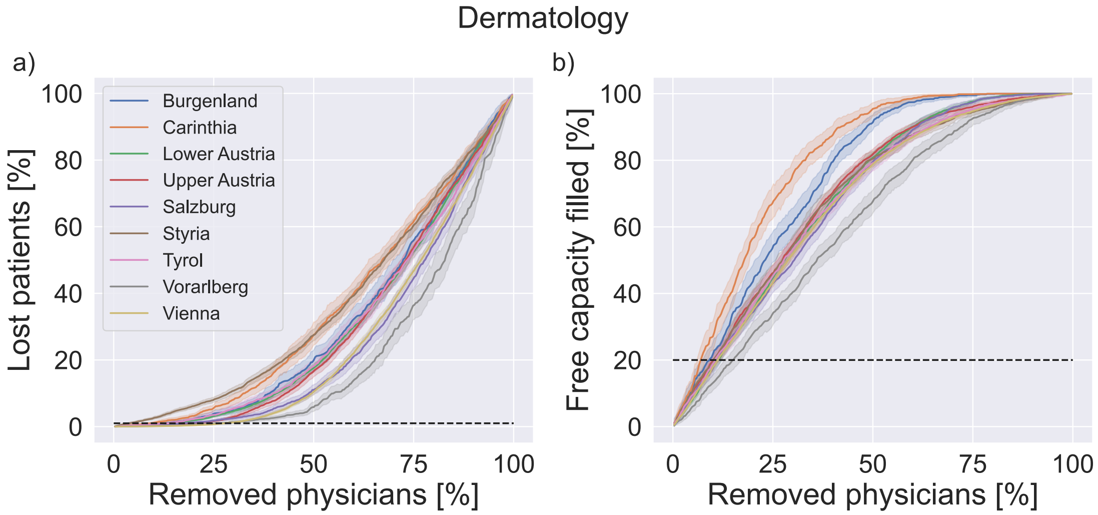

Supplementary Figure 7: Resilience indicators for dermatology. Lost patients and free capacity as a function of removed physicians for dermatologists. Lines and error bands show mean and standard deviation, respectively.

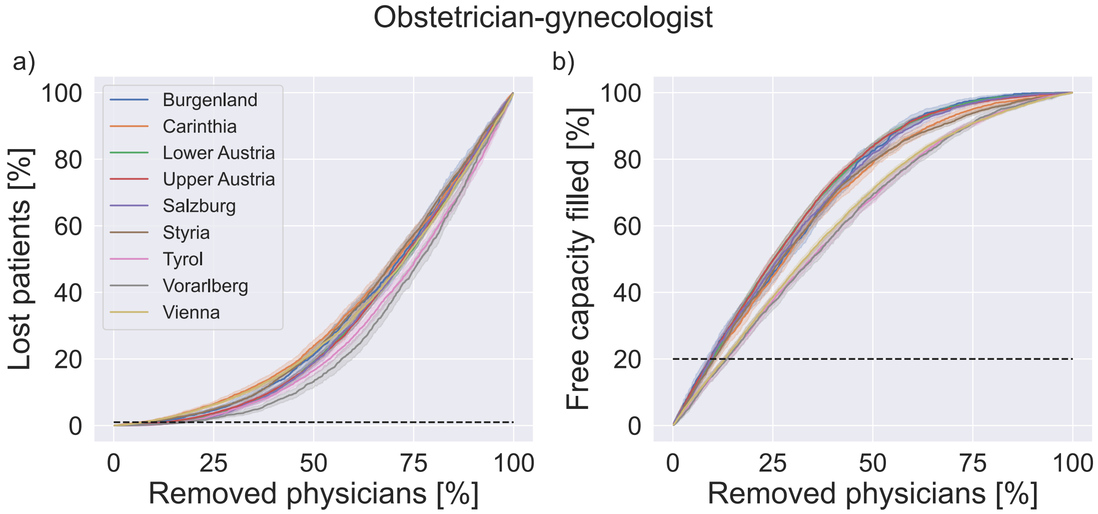

Supplementary Figure 8: Resilience indicators for gynaecology and obstetrics. Lost patients and free capacity as a function of removed physicians for obstetricians/gynecologists. Lines and error bands show mean and standard deviation, respectively.

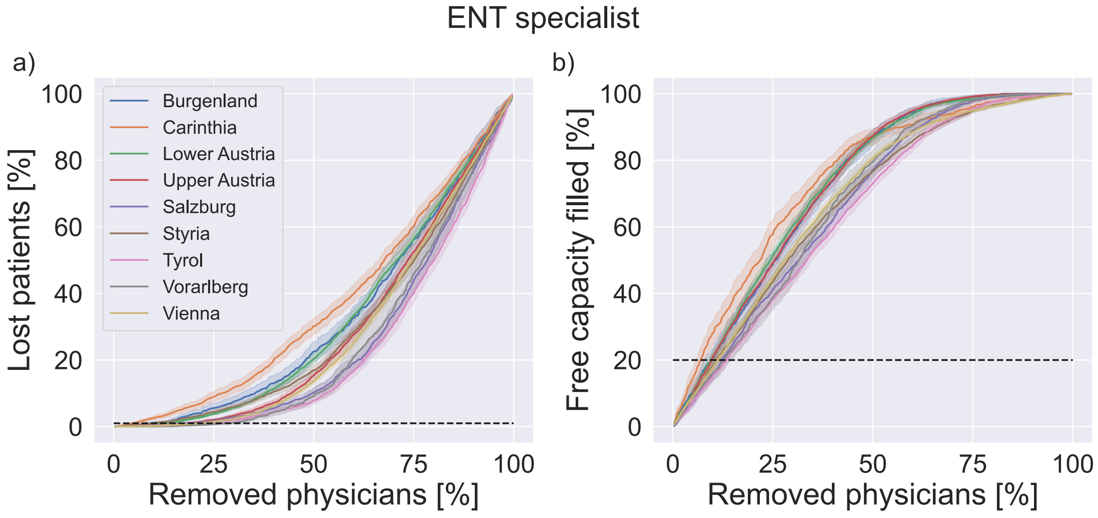

Supplementary Figure 9: Resilience indicators for otorhinolaryngology. Lost patients and free capacity as a function of removed physicians for ENT specialists. Lines and error bands show mean and standard deviation, respectively.

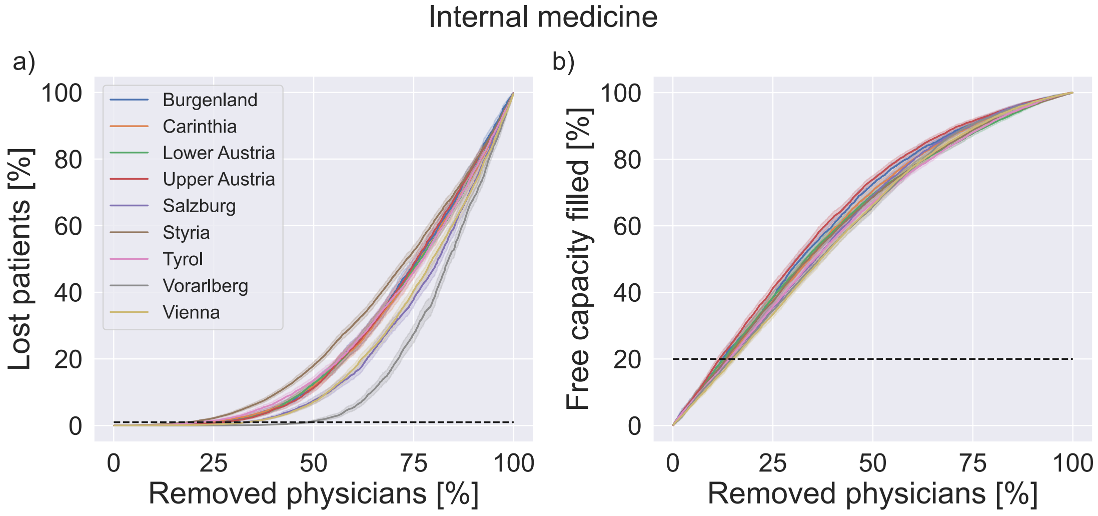

Supplementary Figure 10: Resilience indicators for internal medicine. Lost patients and free capacity as a function of removed physicians for internal medicine. Lines and error bands show mean and standard deviation, respectively.

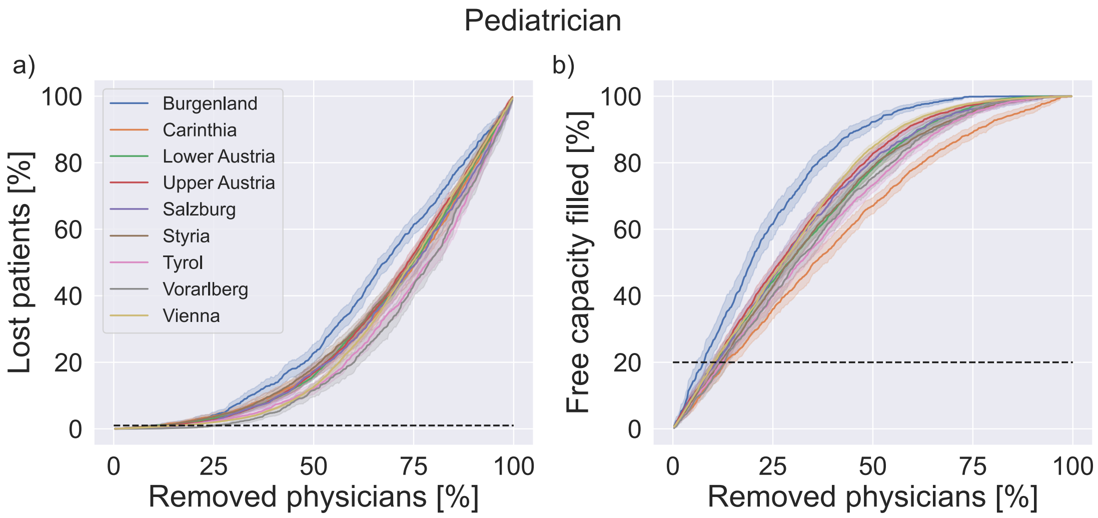

Supplementary Figure 11: Resilience indicators for paediatrics. Lost patients and free capacity as a function of removed physicians for pediatricians. Lines and error bands show mean and standard deviation, respectively.

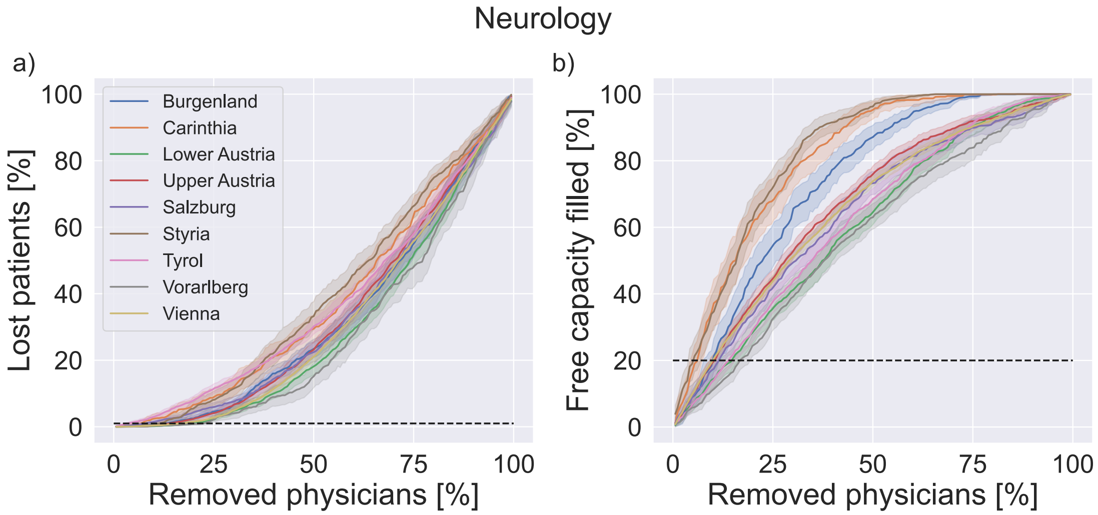

Supplementary Figure 12: Resilience indicators for neurology. Lost patients and free capacity as a function of removed physicians for neurologists. Lines and error bands show mean and standard deviation, respectively.

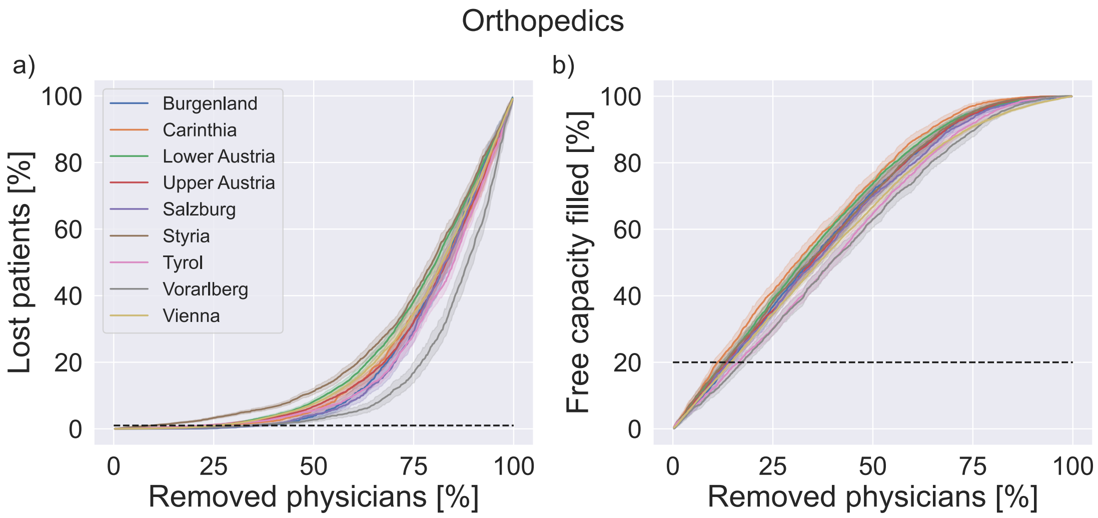

Supplementary Figure 13: Resilience indicators for orthopedics. Lost patients and free capacity as a function of removed physicians for orthopedics. Lines and error bands show mean and standard deviation, respectively.

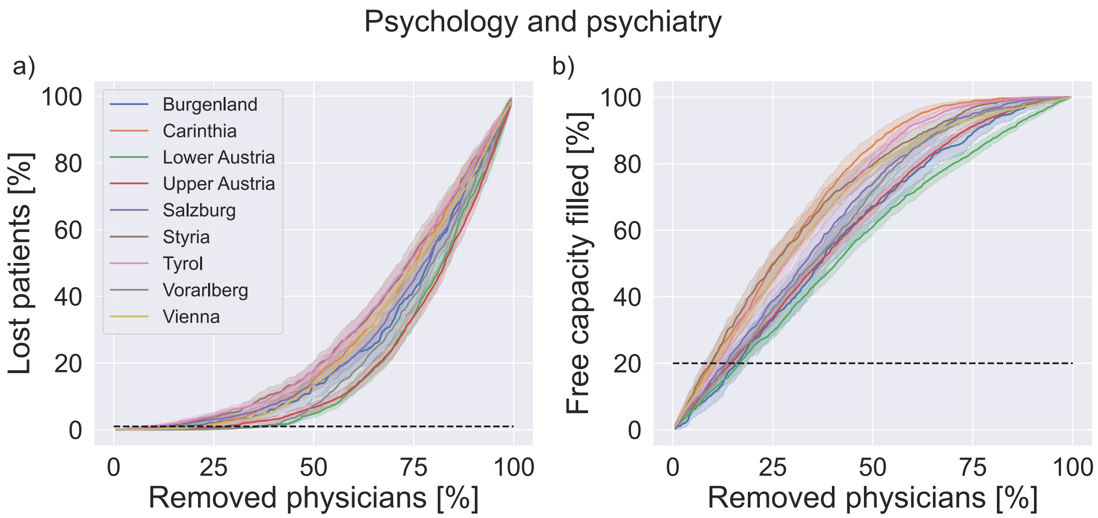

Supplementary Figure 14: Resilience indicators for psychiatry. Lost patients and free capacity as a function of removed physicians for psychiatry. Lines and error bands show mean and standard deviation, respectively.

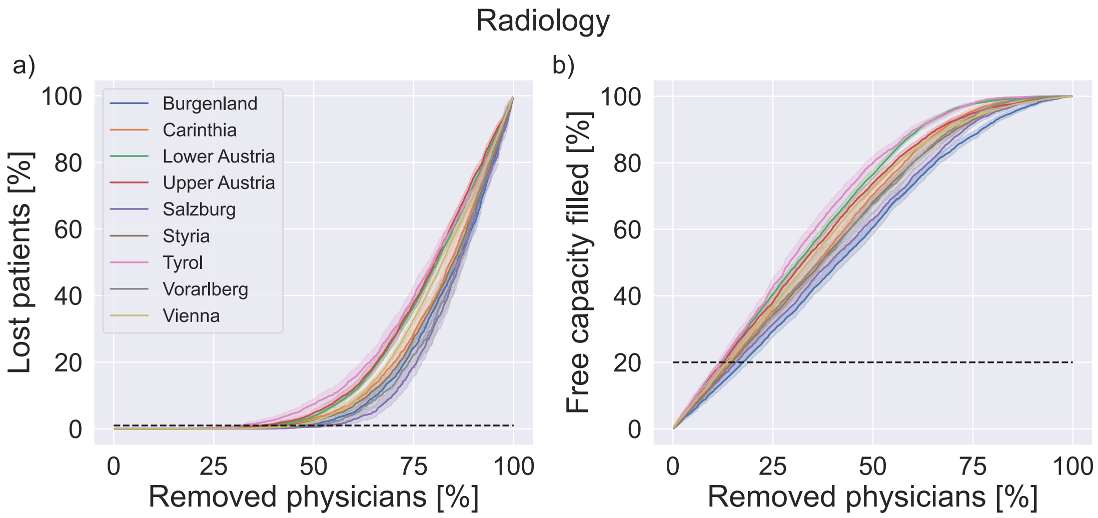

Supplementary Figure 15: Resilience indicators for radiology. Lost patients and free capacity as a function of removed physicians for radiology. Lines and error bands show mean and standard deviation, respectively.

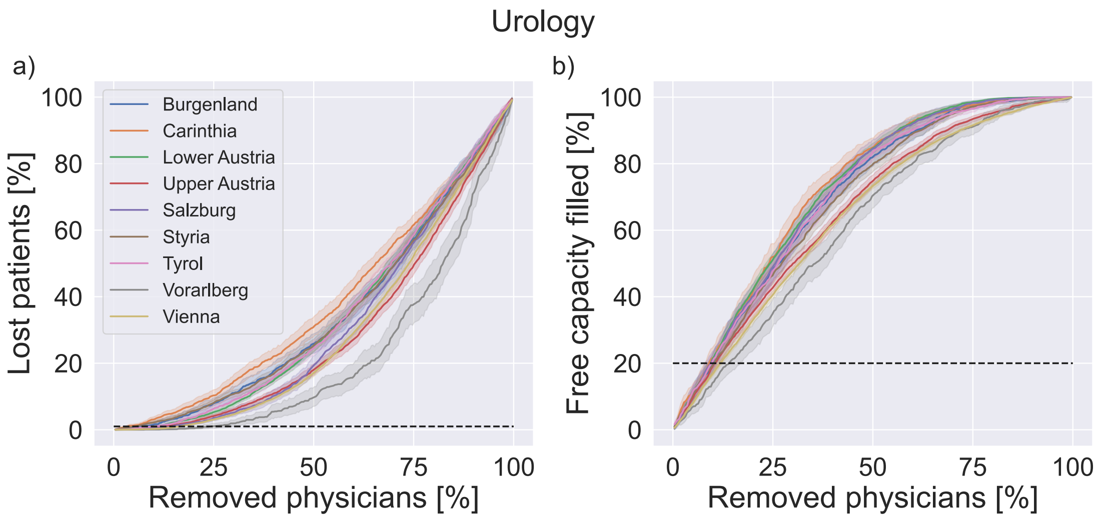

Supplementary Figure 16: Resilience indicators for urology. Lost patients and free capacity as a function of removed physicians for urology. Lines and error bands show mean and standard deviation, respectively.

C Regional resilience indicators - Heat-map table

The following tables 2 and 3 contain the averaged values and standard deviation over all simulation runs for the resilience indicators L_{LP} and L_{FC} for all federal states and specialists as listed in the heat-map figures in the main text.

	Burgenland	Carinthia	Lower Austria	Upper Austria	Salzburg	Styria	Tyrol	Vorarlberg	Vienna
GP	21.0 (2.0)	28.7 (2.8)	17.8 (1.4)	35.3 (2.0)	30.3 (4.0)	20.3 (2.0)	19.4 (3.1)	43.0 (3.4)	56.9 (2.0)
PED	22.0 (13.5)	17.9 (12.1)	14.9 (7.9)	18.6 (9.7)	24.1 (11.1)	13.8 (7.0)	21.4 (9.9)	33.7 (12.0)	17.4 (5.2)
OBGYN	17.9 (10.7)	10.0 (6.5)	16.0 (5.8)	13.7 (4.6)	21.1 (7.6)	10.1 (4.8)	19.6 (7.5)	23.2 (9.8)	8.4 (3.1)
OPH	19.9 (11.3)	17.8 (9.8)	7.2 (4.0)	12.9 (5.4)	12.6 (8.2)	7.0 (5.3)	11.6 (7.4)	27.6 (14.2)	11.9 (3.9)
URO	17.6 (13.2)	11.2 (9.4)	11.7 (6.7)	15.2 (8.1)	22.5 (11.7)	9.4 (7.1)	16.4 (9.0)	47.1 (20.3)	17.6 (4.0)
DER	21.1 (13.5)	16.5 (11.0)	16.6 (6.8)	22.4 (7.3)	23.0 (11.3)	6.9 (5.6)	19.1 (10.1)	37.1 (16.2)	29.0 (5.0)
ENT	22.5 (13.2)	11.1 (8.2)	15.6 (6.7)	21.5 (8.3)	26.8 (12.6)	12.9 (8.0)	26.6 (14.4)	36.6 (14.5)	23.0 (6.0)
IM	30.7 (8.6)	26.4 (7.7)	29.2 (5.9)	30.4 (7.3)	36.1 (7.8)	20.3 (5.5)	25.0 (7.7)	52.9 (7.7)	34.4 (4.5)
SRG	46.9 (12.4)	45.5 (15.3)	35.7 (9.0)	42.9 (14.1)	59.7 (11.5)	13.8 (10.9)	29.4 (15.5)	64.5 (23.9)	33.2 (7.4)
ORTH	43.6 (10.9)	33.9 (13.7)	24.7 (8.2)	25.0 (9.7)	42.1 (12.4)	12.9 (8.6)	27.8 (13.5)	45.3 (17.5)	27.2 (5.6)
NEU	24.6 (14.3)	22.0 (18.2)	25.0 (9.6)	21.1 (10.3)	15.8 (12.2)	25.4 (13.1)	10.2 (7.1)	36.8 (20.0)	18.4 (6.0)
RAD	52.5 (6.8)	45.7 (12.4)	40.5 (6.8)	41.5 (9.4)	61.3 (8.8)	44.4 (8.7)	40.2 (11.5)	59.8 (13.4)	46.5 (4.7)
PSY	44.9 (23.1)	29.7 (14.4)	43.1 (10.4)	38.6 (14.2)	22.1 (11.5)	24.0 (14.2)	19.3 (13.8)	46.1 (14.4)	27.8 (9.3)

Supplementary Table 2: Lost patients indicator. Mean (standard deviation) of resilience indicators L_{LP} of all states and specialties in addition to panel a) of heat-map Fig. 3 in the main text. For a list of abbreviations of specialists see Table 1 in the main text.

	Burgenland	Carinthia	Lower Austria	Upper Austria	Salzburg	Styria	Tyrol	Vorarlberg	Vienna
GP	9.4 (2.1)	9.2 (1.0)	8.6 (0.7)	11.6 (1.0)	9.8 (1.3)	8.9 (1.2)	9.7 (1.0)	12.5 (1.9)	15.0 (0.7)
PED	10.1 (7.0)	15.5 (8.4)	11.1 (4.1)	10.9 (4.9)	13.7 (7.9)	13.1 (5.6)	13.6 (7.2)	15.5 (7.8)	10.8 (3.5)
OBGYN	10.5 (6.1)	10.7 (5.1)	9.9 (3.0)	9.8 (3.1)	10.3 (4.9)	10.4 (3.0)	14.2 (5.0)	14.3 (6.2)	13.2 (2.7)
OPH	10.9 (7.2)	11.7 (7.1)	10.9 (4.2)	11.4 (4.2)	13.2 (5.5)	11.1 (4.1)	13.2 (6.6)	13.7 (7.1)	10.7 (2.8)
URO	11.4 (6.8)	11.3 (8.9)	9.6 (3.3)	11.5 (4.6)	10.8 (7.5)	10.9 (6.0)	11.0 (7.0)	22.0 (11.2)	12.1 (2.8)
DER	11.5 (8.9)	8.2 (5.9)	12.0 (4.1)	10.6 (4.2)	12.4 (6.9)	11.9 (6.2)	12.0 (6.8)	18.1 (10.3)	11.4 (2.9)
ENT	11.9 (7.7)	10.1 (7.1)	9.8 (3.7)	9.9 (4.0)	14.1 (7.0)	11.5 (5.0)	14.0 (5.6)	13.8 (9.2)	11.4 (3.4)
IM	13.6 (5.3)	14.4 (4.6)	13.4 (3.8)	12.3 (3.7)	14.6 (4.7)	13.6 (2.9)	14.4 (3.7)	15.0 (4.2)	15.3 (3.5)
SRG	14.8 (9.2)	16.4 (8.5)	14.9 (5.1)	16.7 (5.2)	17.1 (7.6)	13.0 (7.8)	17.0 (8.9)	22.3 (18.5)	14.9 (4.5)
ORTH	15.1 (7.3)	12.7 (5.7)	13.4 (3.6)	13.9 (3.9)	15.0 (6.4)	14.4 (7.2)	18.1 (7.3)	17.9 (7.4)	14.5 (3.2)
NEU	15.2 (10.6)	11.7 (10.0)	19.2 (11.7)	11.0 (6.0)	14.3 (9.8)	9.8 (8.0)	15.3 (9.4)	21.7 (11.3)	10.3 (3.9)
RAD	18.5 (6.8)	15.4 (6.3)	12.8 (3.2)	13.5 (5.7)	16.9 (7.5)	14.9 (3.8)	13.0 (5.9)	15.6 (8.7)	14.0 (3.0)
PSY	19.7 (13.9)	14.7 (9.4)	18.5 (7.8)	15.5 (5.8)	15.6 (8.8)	12.4 (8.1)	12.4 (7.5)	16.1 (10.1)	11.5 (5.0)

Supplementary Table 3: Free capacity indicator. Mean (standard deviation) of resilience indicators L_{LP} of all states and specialties in addition to panel b) of heat-map Fig. 3 in the main text. For a list of abbreviations of specialists see Table 1 in the main text.

D Interactive online visualisation tool

The tool contains three components (see Supplementary Fig. 17): 1) an overview of resilience indicators per federal state and medical specialty; 2) a detailed view of indicators for the selected specialty in the selected federal state, including risk & benefit scores, information on free capacity & lost patients; 3) the physicians' network within a medical field, displaying indicators per physician.

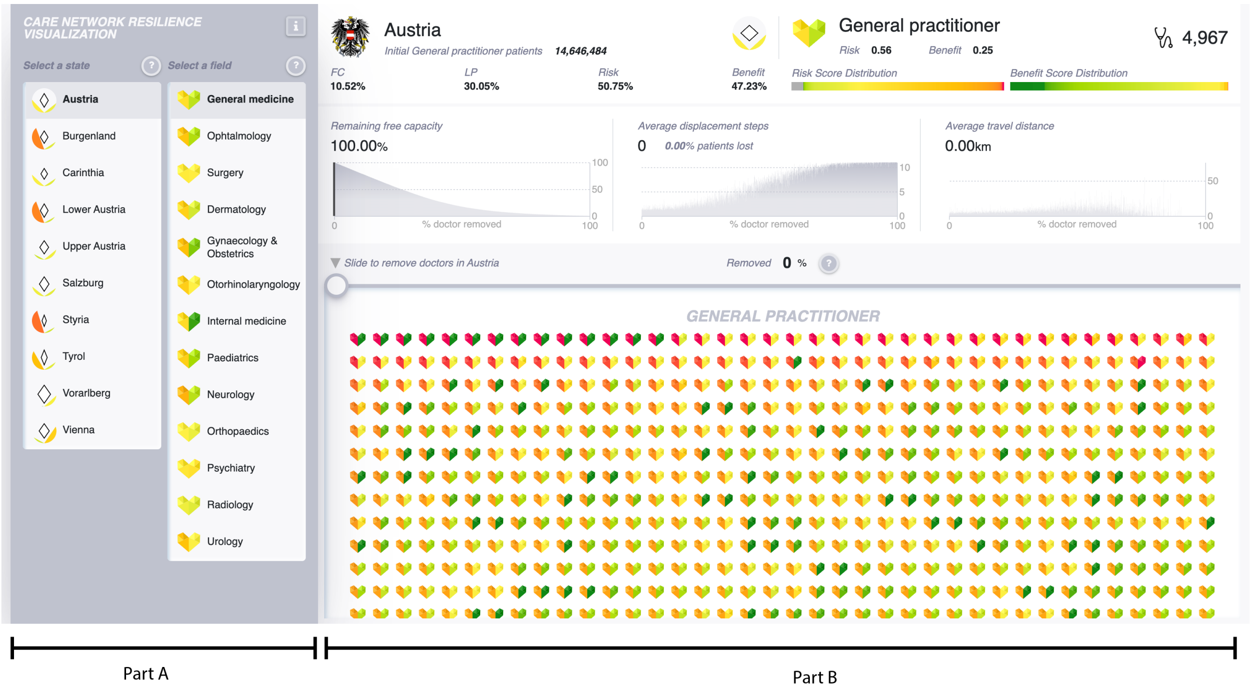

Supplementary Figure 17: Overview of the online tool's interface. A) overview of resilience indicators; B) result view of the selection in A), currently showing detailed indicators and all physicians for general medicine in Austria .

In order to allow the comparison of resilience indicator on an aggregate level, we developed two custom glyphs that respectively summarise the characteristics of each federal state and medical specialty.

To visualise a federal state's resilience, we consider four attributes: free capacity, lost patients, risk level and benefit level. The values of free capacity and lost patients are the average values of all the medical specialties in the federal state. In the glyph shown in Supplementary Fig. 18, free capacity and lost patients values are respectively represented by the length of the diagonals of a rhombus. Increased length represents a higher value, so that the larger area of the rhombus implies a better resilience performance as physicians are removed from the system. At the same time, the dimensions of the rhombus provide visual cues of the proportion of these two attributes. The values of risk and benefit on the federal state level are the percentage of physicians per state with a risk/benefit score that is higher than the nationwide average risk/benefit score. The risk and benefit attributes are respectively represented by a colour-coded filled arc area in a semicircle. Green thereby means a low value, whereas red indicates a high value.

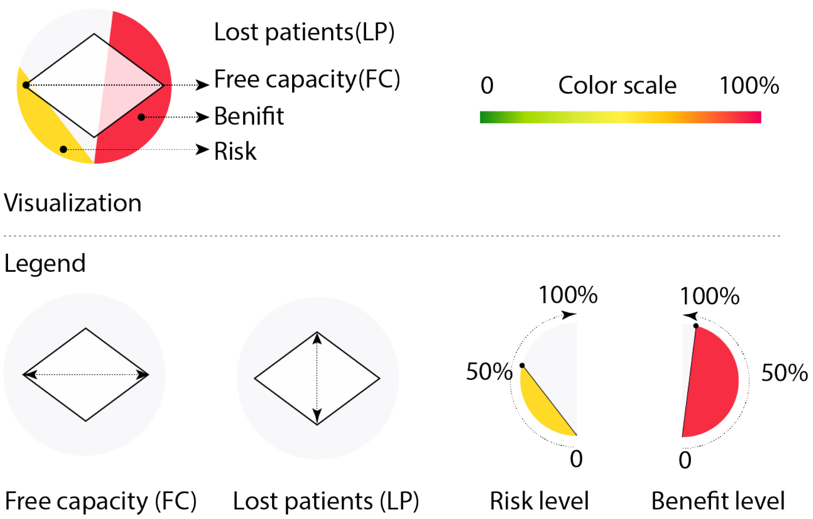

Supplementary Figure 18: State specific glyph. The custom glyph encoding four aggregated indicators per federal state.

To encode the aggregate resilience of a medical specialty, we present the average values of the risk and benefit scores of physicians in a field within the two halves of a heart shaped glyph depicted in Supplementary Fig. 19. We use a similar approach as in the semicircle representation for federal states: the colour of the left half of heart shows the risk score and the right half shows the benefit score.

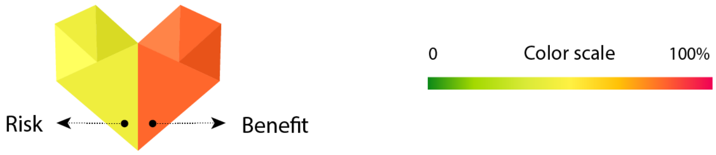

Supplementary Figure 19: Specialty/physician specific glyph. The custom glyph encoding an aggregate of a medical specialty's resilience indicators. The same glyph is used to represent individual physicians.

The overview panel of resilience indicators also serves as a filter for selecting a particular medical field in a particular federal state. After the selection, three sub-components are provided: First, a basic profile that contains four attributes on state level (free capacity, lost patients, risk and benefit levels) and two attributes on specialty level (risk and benefit scores) that are described as an aggregate as well as by their distributions, and finally the number of physicians and patients. The second component shows the model results during a physician removal step (Supplementary Fig. 20). The third component displays the list of physicians in the selected specialty.

The model results contain three charts: remaining free capacity, average displacement steps and average travel distance. The charts visualise the value changes in respect to the percentage of removed physicians. Users can use a slider to adjust the value from "0%" to "100%", in order to

remove a certain amount of physicians (on national level) and compare the values.

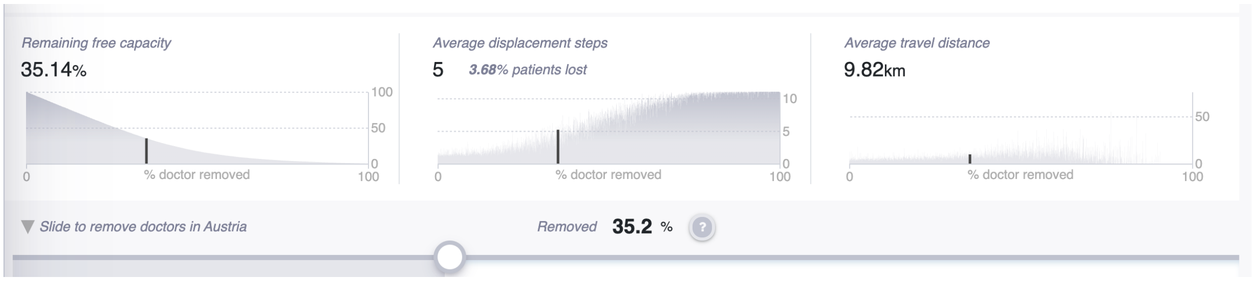

Supplementary Figure 20: Slider charts. The three charts of the physician removal model results indicating values for a physician removal rate of 35.2%. The resulting free capacity is 34.15%, patients take an average of 5 steps and travel 9.82km to find a physician; 3.68% of patients are lost.

Below the model results panel, the list of physicians in the selected field are displayed. Users can click on a physician and view the physician's profile as viewed in Supplementary Fig. 21. The profile displays the physician's state, risk and benefit scores, the number of initial and shared patients, and a snap-view of the physician's network. Clicking the explore button, leads to the third component of the tool: physicians' networks.

The physicians' network component is used to illustrate the displacement steps of a "lost" patient. It assumes that the current chosen physician always turns out to be unavailable, so the user has to choose another physician based on the current physician's network (see Supplementary Fig. 22). We expect the user can learn the fundamentals of this simulation system better by interacting with a visual network.

In the visit history in Supplementary Fig. 23 on the top of the panel, users can view the steps they have made and undo the previous step. Since in our model we also assume that patients

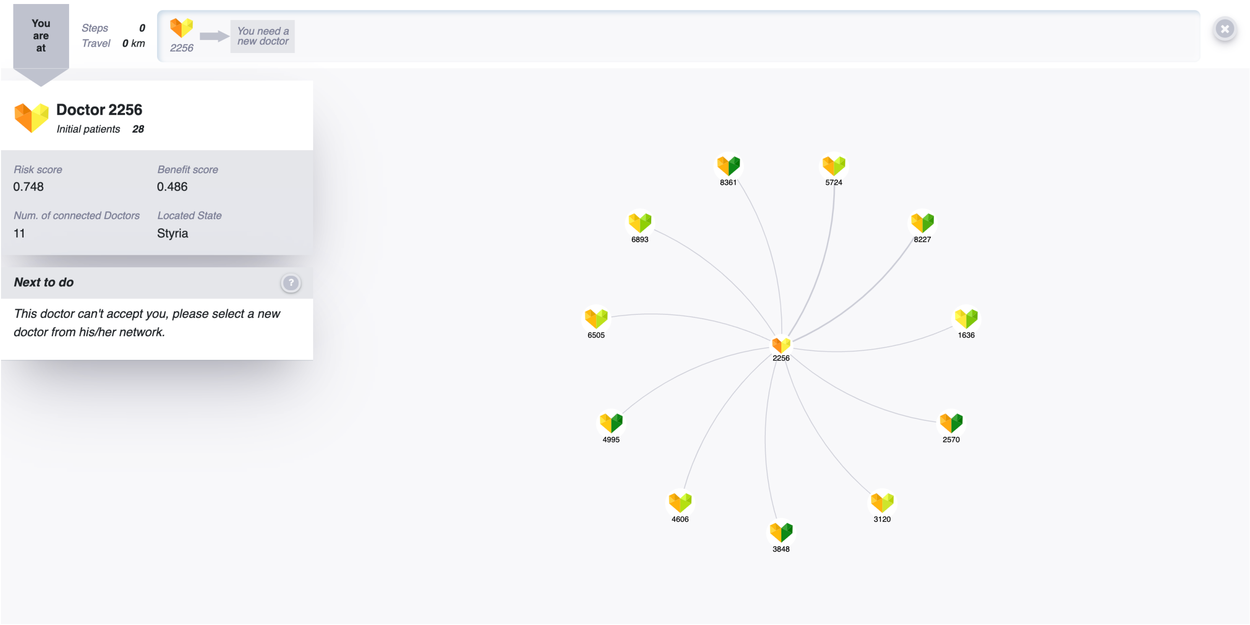

Supplementary Figure 21: Screenshot of the detailed network of a selected physician. Selecting a physician from the list opens a pop-up window on the left, displaying the profile of the selected physician. The strength of the connections can be used to compare how many patients two physicians shared relatively to the other physicians in the network. Detailed values can be retrieved by hovering with the mouse pointer over a connection.

can (and are willing to) travel only a limited distance from their starting location to find a new physician and are only willing to contact a limited number of new physicians, the user cannot select a physician who is more than 100 km away from the starting location of their initial physician, or over 10 steps away in the physician network. As such, the user always plays the role of a "lost" patient in order to learn how patients can become lost in the system.

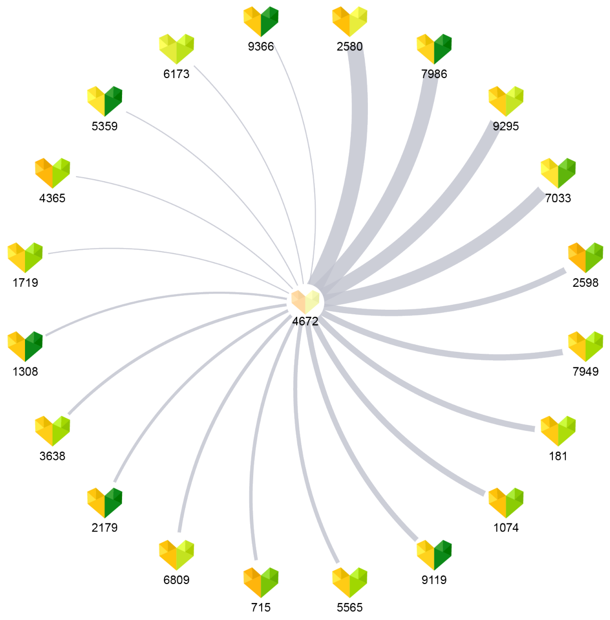

Supplementary Figure 22: Screenshot of a selected physician's network. The nodes represent the connected physicians, i.e., physicians who have shared patients with the current physician(ID: 4672). The width of edges represents the number of shared patients. Wider edges indicate a higher number.

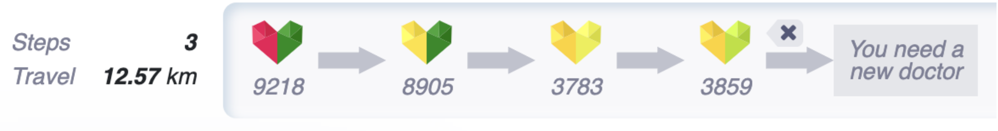

Supplementary Figure 23: Physician visit history. In this screenshot, the user has visited three additional physicians and travelled 12.57 km from the starting position.

E Results for different large-scale shock sizes

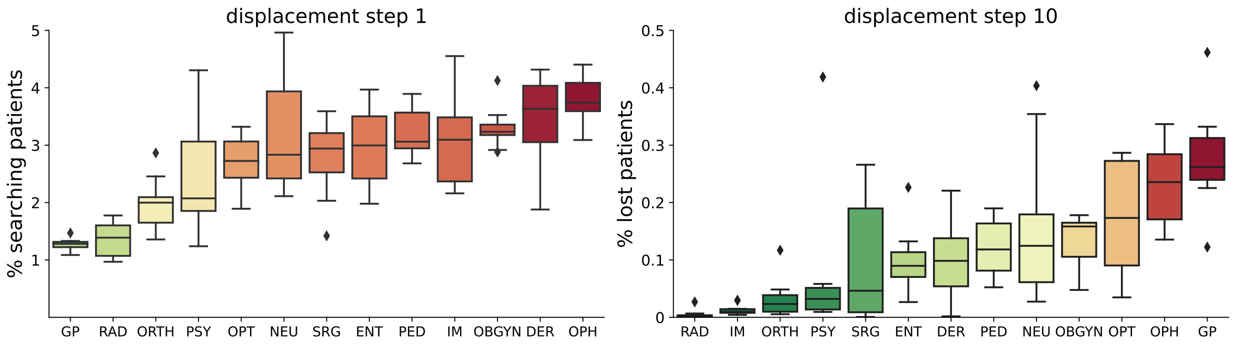

Supplementary Figure 24: Impact of a large-scale unavailability event (7%) on searching and lost patients. Percentage of displaced patients for each specialty one and ten time steps after a large-scale shock where 7% of physicians of a given specialty were removed from the network. Patients that remain displaced after 10 time steps become lost. Boxes show the quartiles of the dataset of an ensemble of 10 simulation runs, whiskers show the rest of the distribution. Points over 1.5 of the inter-quartile range past the high and low quartiles are consideret outliers and shown as individual points. For a list of abbreviations of specialists see Table 1 in the main text.

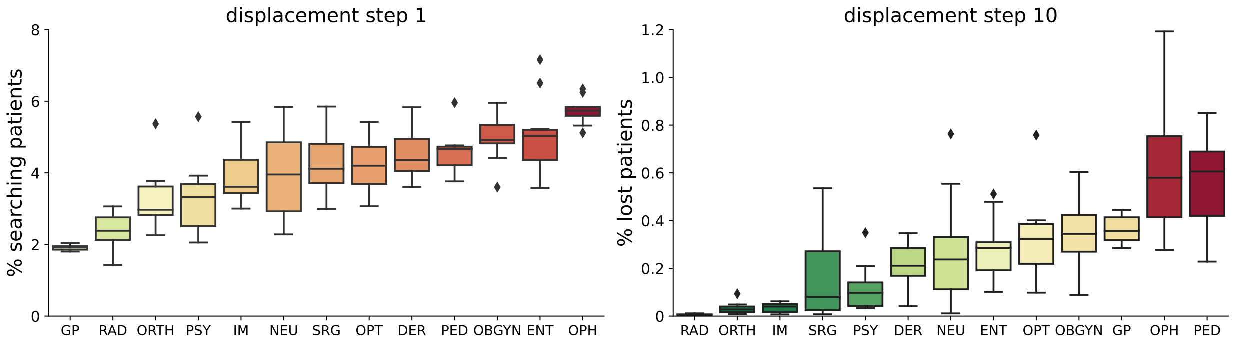

Supplementary Figure 25: Impact of a large-scale unavailability event (10%) on searching and lost patients. Percentage of displaced patients for each specialty one and ten time steps after a large-scale shock where 10% of physicians of a given specialty were removed from the network. Patients that remain displaced after 10 time steps become lost. Boxes show the quartiles of the dataset of an ensemble of 10 simulation runs, whiskers show the rest of the distribution. Points over 1.5 of the inter-quartile range past the high and low quartiles are consideret outliers and shown as individual points. For a list of abbreviations of specialists see Table 1 in the main text.

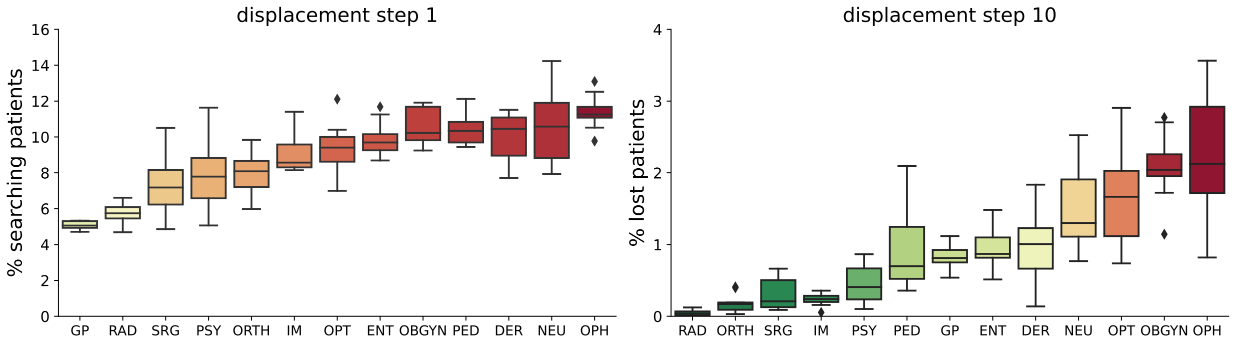

Supplementary Figure 26: Impact of a large-scale unavailability event (20%) on searching and lost patients. Percentage of displaced patients for each specialty one and ten time steps after a large-scale shock where 20% of physicians of a given specialty were removed from the network. Patients that remain displaced after 10 time steps become lost. Boxes show the quartiles of the dataset of an ensemble of 10 simulation runs, whiskers show the rest of the distribution. Points over 1.5 of the inter-quartile range past the high and low quartiles are consideret outliers and shown as individual points. For a list of abbreviations of specialists see Table 1 in the main text.
