# Supplementary material for: Stress-testing the resilience of the Austrian healthcare system using agent-based simulation
Source: Nat Commun. 2022 Jul 23;13:4259. doi: 10.1038/s41467-022-31766-7 (PMC9308034; doi:10.1038/s41467-022-31766-7)
Supplement: Supplementary file 2 — Reporting Summary [file 41467_2022_31766_MOESM2_ESM.pdf]

nature portfolio

Corresponding author(s): Peter Klimek

Last updated by author(s): Jun 29, 2022

Reporting Summary

Nature Portfolio wishes to improve the reproducibility of the work that we publish. This form provides structure for consistency and transparency in reporting. For further information on Nature Portfolio policies, see our Editorial Policies and the Editorial Policy Checklist.

Statistics

For all statistical analyses, confirm that the following items are present in the figure legend, table legend, main text, or Methods section.

n/a	Confirmed	
☐	☒	The exact sample size (*n*) for each experimental group/condition, given as a discrete number and unit of measurement
☒	☐	A statement on whether measurements were taken from distinct samples or whether the same sample was measured repeatedly
☐	☒	The statistical test(s) used AND whether they are one- or two-sided<br>*Only common tests should be described solely by name; describe more complex techniques in the Methods section.*
☐	☒	A description of all covariates tested
☒	☐	A description of any assumptions or corrections, such as tests of normality and adjustment for multiple comparisons
☐	☒	A full description of the statistical parameters including central tendency (e.g. means) or other basic estimates (e.g. regression coefficient) AND variation (e.g. standard deviation) or associated estimates of uncertainty (e.g. confidence intervals)
☐	☒	For null hypothesis testing, the test statistic (e.g. *F*, *t*, *r*) with confidence intervals, effect sizes, degrees of freedom and *P* value noted<br>*Give P values as exact values whenever suitable.*
☒	☐	For Bayesian analysis, information on the choice of priors and Markov chain Monte Carlo settings
☒	☐	For hierarchical and complex designs, identification of the appropriate level for tests and full reporting of outcomes
☐	☒	Estimates of effect sizes (e.g. Cohen's *d*, Pearson's *r*), indicating how they were calculated

Our web collection on statistics for biologists contains articles on many of the points above.

Software and code

Policy information about availability of computer code

Data collection	No software was used to collect the patient-contact dataset. Information about opening hours is publicly available on herold.at and was downloaded in March 2020.
Data analysis	Python 3.8.5 was used to perform the simulations and data analysis. Simulation and analysis code for this study is available under MIT license in the repository at https://doi.org/10.5281/zenodo.6342820.

For manuscripts utilizing custom algorithms or software that are central to the research but not yet described in published literature, software must be made available to editors and reviewers. We strongly encourage code deposition in a community repository (e.g. GitHub). See the Nature Portfolio guidelines for submitting code & software for further information.

Data

Policy information about availability of data

All manuscripts must include a data availability statement. This statement should provide the following information, where applicable:

- Accession codes, unique identifiers, or web links for publicly available datasets
- A description of any restrictions on data availability
- For clinical datasets or third party data, please ensure that the statement adheres to our policy

The simulation data generated in this study have been deposited in OSF under accession code https://doi.org/10.17605/OSF.IO/H5E9A. The raw and processed patient contact data are not available due to privacy laws. The dataset is safeguarded by the Austrian Federal Ministry of Health and made accessible to research institutions under strict data protection regulations. To gain access to this data, researchers have to find individual arrangements with the Austrian Federal Ministry of Health. We publish a sample data set under accession code https://doi.org/10.5281/zenodo.6576023 to showcase how the patient sharing network is created from such data and how simulations are run.The Federal Law on Documentation in the Health Care System in Austria provides the legal basis for written informed

consent not being required for this study: It allows the documentation of health-related data in the intra- and extramural outpatient and inpatient care sectors, as well as for the processing of patients' and service providers' data in pseudonymized form for certain purposes including (long-term) monitoring of epidemiological developments relevant to health policy as well as the implementation and further development of integrated health structure planning and health services research. The opening hour data is publicly available at herold.at. The data collected in March 2020 for this study is available upon reasonable request from the corresponding author. Data can be shared for research purposes. Requests will be considered for 10 years after publication of this article.Data used in this study can be provided by the Austrian National Public Health Institute: Gesundheit Österreich GmbH, Stubenring 6, 1010 Vienna, kontakt@goeg.at. After reviewing the request, data will usually be provided within 4 weeks.

Field-specific reporting

Please select the one below that is the best fit for your research. If you are not sure, read the appropriate sections before making your selection.

☐ Life sciences ☒ Behavioural & social sciences ☐ Ecological, evolutionary & environmental sciences

For a reference copy of the document with all sections, see nature.com/documents/nr-reporting-summary-flat.pdf

Behavioural & social sciences study design

All studies must disclose on these points even when the disclosure is negative.

Study description	This study is a quantitative data re-use study. We use agent based simulations calibrated to Austrian physician-patient contact data to assess the resilience of the Austrian healthcare system.
Research sample	All residents of Austria covered by the universal Austrian social insurance that had at least one contact with a contracted physician in the year 2018. The sample is representative, it includes all residents. There was no sampling involved, the dataset was comprehensive. The dataset is safeguarded by the Austrian Federal Ministry of Health and made accessible to research institutions under strict data protection regulations.
Sampling strategy	There was no sampling involved. The dataset is comprehensive.
Data collection	The data is routinely collected by Austrian health authorities as administrative claims data (containing all outpatient contacts of all contracted physicians) and made available to selected research partners under a strict data protection regulation by the Austrian ministry of health. Data on opening hours was scraped from publicly available websites (herold.at) with permission of the data owners. Blinding of researchers to experimental conditions and the study hypothesis is not applicable to our research design.
Timing	The patient-contact dataset includes information from Jan, 1 2018 until 31, Dec 2018. The opening hours data was collected in March 2020.
Data exclusions	From the opening hour data we exclude non-contracted physicians. We exclude physicians that do not indicate any opening hours. We exclude physicians with specialties that do not correspond to any specialties in the patient-contact data.<br>From the patient-contact data we exclude physician-physician connections that correspond to only a single patient contact. In addition we exclude physician-physician connections with a geographical distance larger than 100km.
Non-participation	Not applicable due to the secondary use of administrative data.
Randomization	Not applicable due to the secondary use of administrative data. There was no sampling involved, the dataset was comprehensive.

Reporting for specific materials, systems and methods

We require information from authors about some types of materials, experimental systems and methods used in many studies. Here, indicate whether each material, system or method listed is relevant to your study. If you are not sure if a list item applies to your research, read the appropriate section before selecting a response.

Materials & experimental systems

n/a	Involved in the study	
☒	☐	Antibodies
☒	☐	Eukaryotic cell lines
☒	☐	Palaeontology and archaeology
☒	☐	Animals and other organisms
☒	☐	Human research participants
☒	☐	Clinical data
☒	☐	Dual use research of concern

Methods

n/a	Involved in the study	
☒	☐	ChIP-seq
☒	☐	Flow cytometry
☒	☐	MRI-based neuroimaging
